# Dual yolk-shell structure of carbon and silica-coated silicon for high-performance lithium-ion batteries

**DOI:** 10.1038/srep10908

**Published:** 2015-06-03

**Authors:** L. Y. Yang, H. Z. Li, J. Liu, Z. Q. Sun, S. S. Tang, M. Lei

**Affiliations:** 1 School of Materials Science and Engineering, Central South University, Changsha, Hunan 410083, China; 2 Education Ministry Key Laboratory of Non-ferrous Materials Science and Engineering, Central South University, Changsha, Hunan 410083, PR China; 3 Institute for Superconducting and Electronic Materials, University of Wollongong, Innovation Campus, North Wollongong, NSW 2500, Australia; 4 State Key Laboratory of Information Photonics and Optical Communications, Beijing University of Posts and Telecommunications, Beijing 100876, China

## Abstract

**Silicon batteries have attracted much attention in recent years due to their high theoretical capacity, although a rapid capacity fade is normally observed, attributed mainly to volume expansion during lithiation. Here, we report for the first time successful synthesis of Si/void/SiO**_**2**_**/void/C nanostructures. The synthesis strategy only involves selective etching of SiO**_**2**_ **in Si/SiO**_**2**_**/C structures with hydrofluoric acid solution. Compared with reported results, such novel structures include a hard SiO**_**2**_**-coated layer, a conductive carbon-coated layer, and two internal void spaces. In the structures, the carbon can enhance conductivity, the SiO**_**2**_ **layer has mechanically strong qualities, and the two internal void spaces can confine and accommodate volume expansion of silicon during lithiation. Therefore, these specially designed dual yolk-shell structures exhibit a stable and high capacity of 956** **mA h g^−1^ after 430 cycles with capacity retention of 83%, while the capacity of Si/C core-shell structures rapidly decreases in the first ten cycles under the same experimental conditions. The novel dual yolk-shell structures developed for Si can also be extended to other battery materials that undergo large volume changes.**

Recently, the rapidly rising price of petroleum and growing concerns about global warming have brought a great deal of attention to lithium-ion batteries with high capacity and energy density for future electric vehicles and portable electronics^1^,^2^,^3^,^4^,^5^,^6^. Nevertheless, the performance of currently commercialized lithium-ion batteries must be further improved to meet the increasing demand for high energy storage capacity. Therefore, novel electrode materials with higher capacity and higher power density are urgently needed.

Among the various anode materials, silicon is one of the most promising candidates due to its high theoretical capacity (~3580 mA h g^−1^, Li_15_Si_4_) among alloy type anode materials and relatively low discharge potential (~0.4 V vs. Li/Li^+^)^4^,^5^. Despite these advantages, silicon anodes have two major disadvantages that have prevented their widespread use. First, the large volume changes (~300%) in silicon upon insertion and extraction of lithium-ions lead to severe electrode pulverization, which results in the loss of contact between the active materials and the current collector, leading to rapid capacity fading. Second, the continual pulverization of silicon during cycling causes the electrode surface to be cyclically exposed to the electrolyte. This generates continual formation of solid-electrolyte interphase (SEI) films, contributing to capacity fading and poor coulombic efficiency.

In an attempt to overcome these limitations of silicon, much attention has been devoted to the design and fabrication of silicon nanostructures, such as silicon nanowires^6^,^7^,^8^ and nanotubes^9^,^10^, three-dimensional (3D) porous silicon^11^,^12^,^13^,^14^, and silicon in composites with carbon or oxides^15^,^16^,^17^,^18^,^19^,^20^. Encouraging results have been achieved through these efforts. In particular, Si/SiO_*x*_ composites^21^,^22^, Si/SiO_2_/C^23^,^24^,^25^, and yolk-shell structured Si/C^26^,^27^,^28^ have demonstrated excellent electrochemical performance. These performances can be ascribed to the C or SiO_*x*_ shell on the outside of the silicon, which can offer a static surface for the formation of a thin and stable SEI, preserving the anode from irreversible reaction with the electrolyte^29^,^30^,^31^. Additionally, the existing hollow structures in the yolk-shell strucure can provide extra space for the volume expansion of silicon, which guarantees the structural integrity of the electrode^29^,^30^,^31^. These distinguishing features provide enlightened guidance for nanostructured design of high-performance silicon batteries.

In this paper, we uniformly coated silicon with a silica layer via the Stöber method and then coated Si/SiO_2_ composites with a carbon layer. Taking advantage of the inhomogeneous nature of silica shells prepared by the Stöber method^32^, proper etching conditions were chosen for selectively etching the SiO_2_ shell by means of a hydrofluoric acid (HF) treatment. By this process, a small portion of the outer layer and a large portion of the interior layer of the SiO_2_ shell were removed. The novel Si/void/SiO_2_/void/C structures were obtained. The advantages of dual yolk-shell silicon structures lie in the presence of internal void spaces and the mechanically strong SiO_2_ layer, which limits the degree of volume expansion of silicon during lithiation. In addition, the amorphous SiO_2_ and C intrinsically have advantages as shell materials due to their chemical inertness, porous structure, and size-selective permeability^33^,^34^,^35^,^36^,^37^. They can provide a double barrier to prevent the electrolyte from reaching the surface of the silicon nanoparticles and protect the anode from subsequent irreversible reaction with the electrolyte^31^. With the help of the dual yolk-shell structures, the capacity of the silicon half-cell could be stabilized at 956 mA h g^−1^ at 0.46 A g^−1^ after 430 cycles with capacity retention of 83%, while the capacity of the Si/C core-shell structures rapidly decreases in the first ten cycles under the same experimental conditions. Therefore, such dual yolk-shell structures can also be extended to other battery materials that undergo large volume changes.

## Results

**Figure 1:**
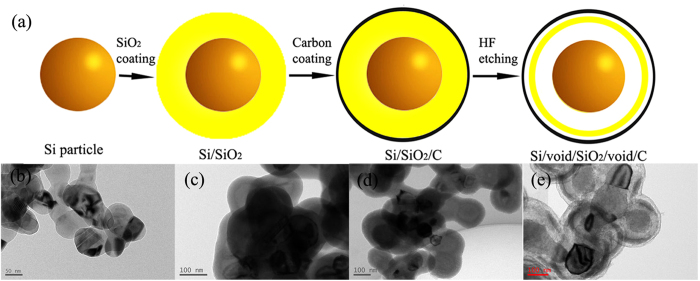
(**a**) Schematic illustration of the fabrication process for the dual yolk-shell structure. (**b**), (**c**), (**d**), and (**e**) Corresponding TEM images of Si, Si/SiO_2_, Si/SiO_2_/C, and Si/void/SiO_2_/void/C spheres.

The schematic flowchart in Fig. 1 illustrates the major process steps employed in the present work. A SiO_2_ layer was first coated on Si seeds using the Stöber method. The carbon layer was then obtained through a pyrolytic decomposition of polyvinylidene difluoride (PVDF), which had been already coated on the Si/SiO_2_ nanoparticles. Finally, Si/void/SiO_2_/void/C nanostructures could be prepared by selectively etching the SiO_2_ layer using an HF solution with a proper concentration. The corresponding transmission electron microscope (TEM) images are presented in Fig. 1b,c,d,e. Silica has been frequently used as a source of shell materials^33^,^34^,^35^,^36^, and the Stöber method has attracted much interest for the scalable fabrication of silica shells on nanoparticles via the facile hydrolysis of tetraethyl orthosilicate (TEOS). In addition, the SiO_2_ layer has some unique chemical properties. For instance, Chen’s group has demonstrated that the silica shell on nanoparticles formed by the Stöber method is intrinsically inhomogeneous. The outer layer of the shell is chemically more robust than the inner layer. They obtained multiple yolk-shell nanostructures through selectively etching the silica layer with hot water^32^. Also, Yin’s group used only TEOS to generate core-shell structures, and they showed that polyvinyl pyrrolidone (PVP) could protect the outside of the silica layer to allow selective etching of the inner section^38^. These properties can be exploited for novel synthetic control of silica nanostructures. In our paper, the SiO_2_ layer remains inhomogeneous after the coating with the carbon layer. Therefore, a thin layer (about 4–6 nm) on the robust outside of the silica, and at the same time, a thick layer (about 10–30 nm) on the soft inside of the silica can be etched away by the HF solution.

**Figure 2:**
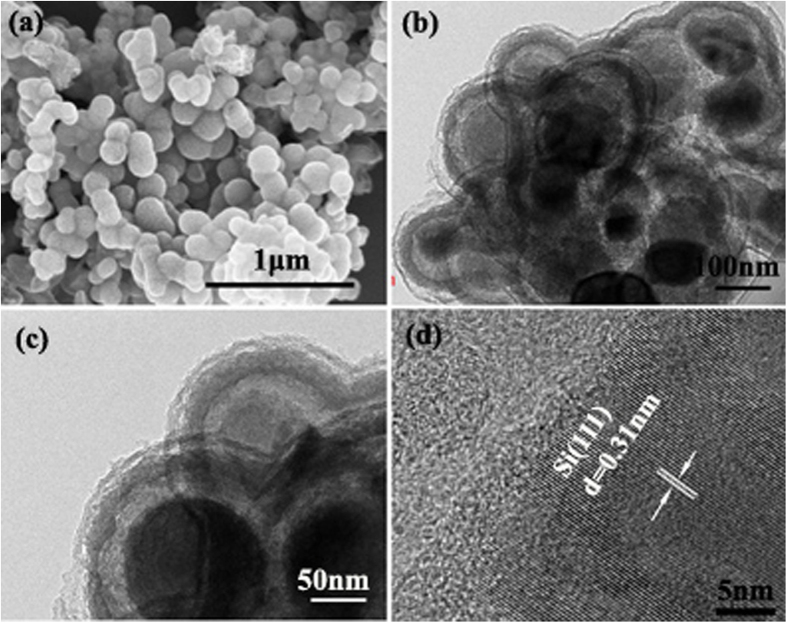
(**a**) SEM, (**b**)TEM, (**c**) magnified TEM, and (**d**) HRTEM images of Si/void/SiO_2_/void/C composite spheres.

Figure 2 shows scanning electron microscope (SEM) images of the dual yolk-shell Si nanocomposite spheres, which exhibit an average diameter of 190 (±10) nm. TEM and high resolution TEM (HRTEM) (Fig. 2b,c, d) observations confirm that dual yolk-shell silicon structures with two hollow buffers and two amorphous shells were obtained.

**Figure 3:**
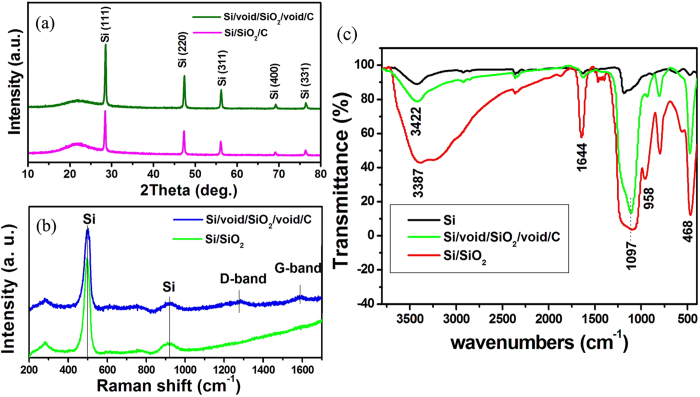
(**a**) XRD patterns of Si/SiO_2_/C composites before and after HF treatment.(**b**) Raman spectra of Si/SiO_2_ and Si/void/SiO_2_/void/C composites. (**c**) FTIR spectra of Si, Si/SiO_2_, and Si/void/SiO_2_/void/C composite.

The X-ray diffraction (XRD) patterns of the Si/SiO_2_/C nanocomposite before and after the selective etching process with HF solution are shown in Fig. 3a. The diffraction peaks appearing at 28.4, 47.3, 56.1, 69.1, and 76.3° can be indexed respectively to the (111), (220), (311), (400), and (331) planes of Si crystallites (ICDD JCPDS no. 27-1402). A broad peak appearing at around 22° indicates the amorphous character of the silica shell. After the selective etching of the silica, a drop in the intensity ratio of amorphous SiO_2_ to Si is clearly observable, indicating the decreased amount of SiO_2_. Figure 3b shows the Raman spectra of the Si/SiO_2_ and Si/void/SiO_2_/void/C nanocomposites. It can be seen that due to the presence of Si nanoparticles, these composites exhibit Si peaks at around 500 cm^−1^ and 918 cm^−1^ 39. The observation of two peaks at 1282 cm^−1^ (known as the D band) and 1591 cm^−1^ (known as the G band)^25^,^40^ are characteristic of the presence of carbon materials in Si/void/SiO_2_/void/C samples.

The Fourier transform infrared spectroscopy (FTIR) spectra in Fig. 3c show the characteristic transmittances of Si, Si/SiO_2_, and Si/void/SiO_2_/void/C powders. Si/SiO_2_ nanocomposite shows peaks at 3387 cm^−1^, 1644 cm^−1^, 1097 cm^−1^, 958 cm^−1^, and 468 cm^−1^, corresponding to –OH, –OH, Si-O, Si-OH, and Si-O-Si stretching, respectively^25^,^41^. The peaks at 3387 cm^−1^ and 1644 cm^−1^ in the spectrum of the Si/void/SiO_2_/void/C powders are reduced, which can be attributed to the decomposition of the –OH band during the carbonization to produce the carbon coating on Si/SiO_2_. The presence of Si-O and Si-O-Si in the products demonstrates the formation of SiO_2_ in the Si/SiO_2_ and Si/void/SiO_2_/void/C nanocomposites.

**Figure 4:**
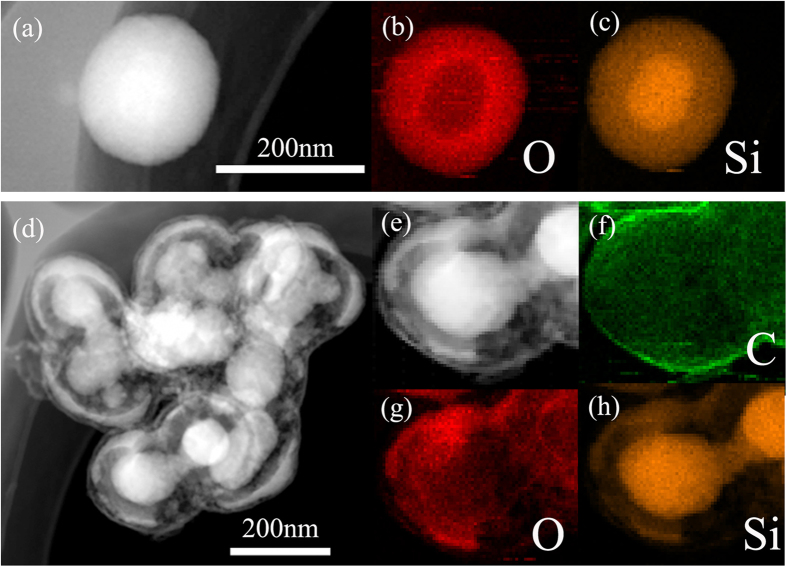
(**a**) STEM image of Si/SiO_2_.(**b**), (**c**) corresponding EDX mapping images of O (red) and Si (orange). (**d**, **e**) STEM image of Si/void/SiO_2_/void/C. (**f**), (**g**), (**h**) corresponding EDX mapping images of C (green), O (red), and Si (orange).

Further evidence for Si/SiO_2_ and Si/void/SiO_2_/void/C nanostructures was identified by dark-field scanning transmission electron microscopy (STEM) images and energy dispersive X-ray (EDX) element mappings (Fig. 4). The SiO_2_ coating can be distinguished in the Si/SiO_2_ nanostructures from the O and Si mappings. The void space between Si and SiO_2_ in the Si/void/SiO_2_/void/C nanostructures can be seen from the Si and O mappings, and the void space between C and SiO_2_ can be seen in the STEM image. The TEM, XRD, Raman, and FTIR investigations, as well as the element mapping data, indicate that the nanocomposites consist of a silicon core, a shell of amorphous SiO_2_, two void spaces, and a carbon layer in the Si/void/SiO_2_/void/C structures.

**Figure 5:**
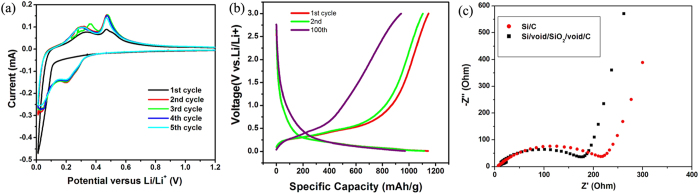
(**a**) Cyclic voltammograms (CVs) from the first 5 cycles for Si/void/SiO_2_/void/C from 0.01 V to 3 V (with only 0.01–1.2 V shown) at a scan rate of 0.05 mV s^−1^.(**b**) Charge and discharge voltage profiles of Si/void/SiO_2_/void/C composite for the 1^st^, 2^nd^, and 100^th^ cycles tested between 0.01 and 3 V at a rate of 0.46 A g^−1^. (**c**) EIS results for Si/void/SiO_2_/void/C and Si/C composites.

**Figure 6:**
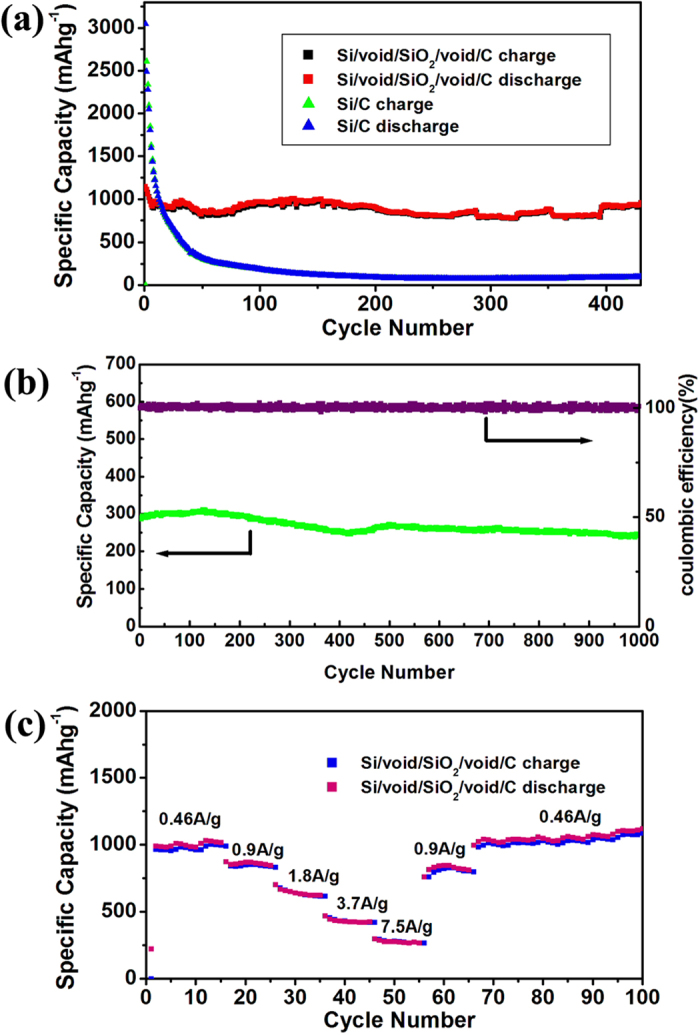
(**a**) Cycling behavior of Si/void/SiO_2_/void/C and Si/C composites at a current density of 0.46 A g^−1^.(**b**) Cycling performance and coulombic efficiency of Si/void/SiO_2_/void/C at a current density of 5.8 A g^−1^. (**c**) Rate capability of Si/void/SiO_2_/void/C composite at different current densities.

To test the electrochemical performance of the dual yolk-shell Si nanocomposite, a two-electrode coin cell using the nanocomposite as the electrode material and lithium metal as counter electrode was fabricated. Cyclic voltammetry experiments (Fig. 5a) show lithiation and delithiation peaks at potentials typical of the reactions of Si^42^. The peak at 0.19 V in the cathodic branch (lithiation) corresponds to the conversion of amorphous Si to Li_*x*_Si. In the anodic branch (delithiation), the two peaks at 0.31 V and 0.47 V are attributed to the delithiation of Li_*x*_Si back to amorphous Si^43^. No SEI formation peak (0.34–0.36 V)^44^ can be observed in the first cycle in the CV curves. Moreover, an activation process occurs, as indicated by the increase in the CV peak intensity. Similar phenomena have been observed elsewhere^8^,^26^,^44^,^45^. The phenomena can be ascribed to the presence of C or an oxide shell outside of the silicon, which can offer a static surface for the formation of a thin and stable SEI, preserving the anode from irreversible reactions with the electrolyte^29^,^30^,^31^,^44^. In addition, in the work of Li’s group^44^, SEI formation on hollow carbon nanospheres/silicon/alumina core-shell structures was not observed, because the insulating Al_2_O_3_ layer suppresses electron transfer from Si to the electrolyte. The decomposition of LiPF_6_ to form LiF and PF_5_ is the only possible mechanism that can occur^44^. Thus only a small amount of LiF is formed due to the decomposition of LiPF_6_ with heat and moisture. Since this process does not involve Li^+^, this may not appear in the CV curves, unlike conventional SEI formation on Si surfaces^44^. Their work provides a new understanding of the mechanism of SEI formation. In our work, the formation of the SEI film on C and oxide shells may be a slow process during the first few cycles, so that the SEI formation peaks were not obvious in the first few cycles in the CV curves. Another possibility is that there is no SEI formation during the cycling, because the insulating SiO_2_ layer has the same effect as the Al_2_O_3_ layer in the work of Li’s group. In the activation process, it may take a few cycles for the outer layers of SiO_2_ and C to become ionically conductive, allowing each Si particle to become lithiated. The activation process can also be observed in our cycling performance in Fig. 6b, as the capacity increases in the first few cycles. Figure 5b shows the 1^st^, 2^nd^, and 100^th^ voltage profiles in the charge and discharge processes in the potential window of 0.01–3 V vs Li/Li^+^. The first cycle at a current rate of 0.46 A g^−1^ shows charge and discharge capacity of 1147 and 1143 mA h g^−1^, respectively. After 2 and 100 cycles, the capacities are 1113 mA h g^−1^ and 968 mA h g^−1^, respectively. The electrochemical impedance spectroscopy (EIS) technique was also utilized to clarify the electrochemical performance of the dual yolk-shell Si nanocomposite compared with Si/C nanocomposite^46^. The charge-transfer resistance parameters of the Si/C electrode are obviously larger than those of the Si/void/SiO_2_/void/C nanocomposite electrode (Fig. 5c).

The Si/void/SiO_2_/void/C electrode displays stable cycling performance and maintains a reversible discharge capacity of 956 mA h g^−1^ after 430 cycles at current density of 0.46 A g^−1^ (Fig. 6a), which is 2.5 times higher than the theoretical capacity of graphite. At the same time, Si/C nanocomposite was also tested in lithium-ion batteries as comparison samples. The initial capacity of Si/C nanocomposite reaches up to 3150 mA h g^−1^ at current density of 0.46 A g^−1^, although a rapid capacity fade is observed upon further cycling. This can be attributed to the expansion/contraction of silicon during the charge and discharge, which results in the pulverization and disruption of the microstructure of the electrode. Figure 6b presents the long-term cycling performance at a charge/discharge rate of 5.8 A g^−1^, which shows stable capacity around 250 mA h g^−1^. Capacity degradation is almost negligible, demonstrating the good stability of the dual yolk-shell silicon structures. Figure 6c shows the charge/discharge capacity at different current densities. Capacity above 950, 830, 610, 410, and 260 mA h g^−1^ is retained at current density of 0.46, 0.9, 1.8, 3.7, and 7.5 A g^−1^, respectively, and when the current density is changed back to 0.46 A g^−1^, the specific capacity recovers to 1000 mA h g^−1^.

From Table 1, it can be seen that although our sample shows worse cyclability than the Si-C yolk shell structure, it is better than those of Si-SiO_x_ core-shell nanowire, Si-SiO_2_ core@shell nanowire, silicon@carbon hollow core–shell, Si@SiO_x_/C nanocomposite, and silicon@TiO_2-x_/carbon microfiber. Considering that the loading of active materials for coin cell testing is about 1.5 mg cm^−2^ and the thickness of the active materials is 200 μm, our sample indeed shows excellent performance as anode material for lithium ion batteries.

## Discussion

The improved cyclability could be ascribed to the characteristics of such dual yolk-shell structures. First, the void spaces in the dual yolk-shell structures allow for some volume expansion of silicon. At the same time, the SiO_2_ layer is mechanically strong and can successfully prevent the Si from expanding, while still allowing lithium ions to pass through. Secondly, the SiO_2_ layer combined with the outside C layer could provide double protection for the anode from irreversible reaction with the electrolyte, and a stable SEI could be built on the outside surface. As a result, these dual yolk-shell silicon structures show stable cyclability.

In the Si/void/SiO_2_/void/C nanocomposite, the electron and ion transport pathways may be a problem due to the void spaces. Numerous nanocomposites containing voids, however, have been reported for use in lithium-ion batteries. We suppose that the electrons and ions are transported through the contact points between the core and SiO_2_ shell, as well as between the SiO_2_ shell and the C shell in our work. So, the decrease in the capacity at large current density (Fig. 6) can be partly ascribed to the slow diffusion of Li^+^ in the Si/void/SiO_2_/void/C structures.

**Figure 7:**
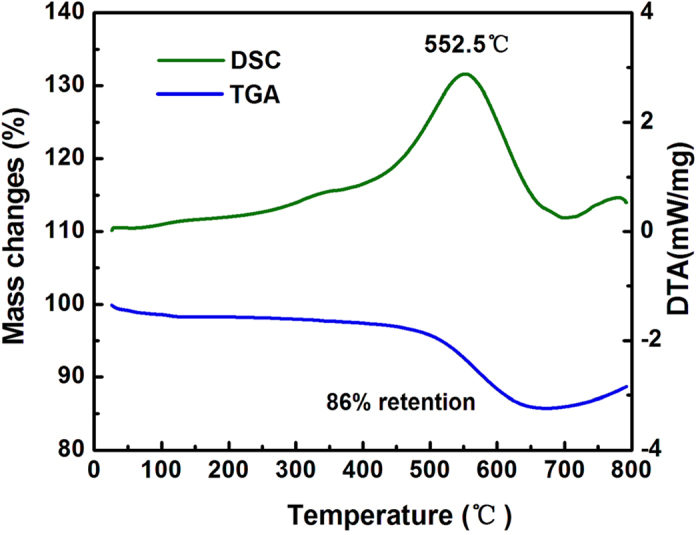


Thermogravimetric analysis (TGA) of the dual yolk-shell silicon structures was carried out in air atmosphere from 30 °C to 800 °C (Fig. 7). The weight content of carbon is about 14%. It is assumed that there was no loss of silicon during the whole synthesis process of the Si/void/SiO_2_/void/C nanocomposite (since losses were avoided as far as possible in the process of collecting products in each step, and in addition, Si is inert compared to SiO_2_ in the reaction with HF). Therefore, the weight content of silicon can be estimated by the data recorded in each step. The weight ratio of silicon in the Si/void/SiO_2_/void/C nanocomposite is about 64%, and that of silica is about 22%.

## Methods

### Material preparation

Si/void/ SiO_2_/void/C nanospheres were synthesized in three steps. Firstly, 0.4 g silicon nanoparticles (with an average diameter of ~100 nm) were dispersed in a mixture of 74 ml ethanol and 10 ml water by ultrasonication. Then 0.15 g polyvinylpyrrolidone (PVP) and 3 ml ammonia water (30%) were added into this solution. 6 ml tetraethyl orthosilicate (TEOS) was added dropwise into the solution under vigorous stirring, and the reaction was incubated at room temperature under stirring for 1 h. The resulting Si/SiO_2_ nanoparticles were isolated by centrifugation. Secondly, polyvinylidene fluoride (PVDF) powder was dissolved in N-methyl-2-pyrrolidinone (NMP) in a weight ratio of 6%^18^. Then, the Si/SiO_2_ powders were added into the solution and stirred for 24 h. The obtained suspension was dried at 90 °C under vacuum for 48 h to vaporize the NMP solvent. The dried substance was transferred into a furnace and pyrolyzed at 650 °C under Ar atmosphere for 2 h to obtain Si/SiO_2_/C composite. Then, the Si/SiO_2_/C nanoparticles were milled in an agate mortar and sieved for subsequent use. Finally, the obtained Si/SiO_2_/C composite was immersed in 0.8 M HF solution for 1 h to selectively etch the SiO_2_ layer. The resulting solid was collected after washing with water four times by centrifugation. Dual yolk-shell structures were obtained.

### Characterization

The morphology and diameter of the Si/void/SiO_2_/void/C nanospheres were characterized with a scanning electron microscope (SEM, FEI Nova Nano SEM 230) and a transmission electron microscope (TEM, JEOLJEM-2100F). Powder X-ray diffraction (XRD) patterns were obtained using an X-ray diffractometer (XRD, Rigaku D/max 2500 XRD with Cu-Kα radiation, *λ* = 1.54178 Å). The amount of C in the Si/void/SiO_2_/void/C nanoparticles was confirmed by a combined differential scanning calorimetry (DSC) and thermogravimetric analysis (TGA) instrument (SDT, Q600) in air atmosphere with a heating rate of 5 °C/min. Raman (LabRam HR-800) and Fourier transform infrared spectroscopy (FTIR) (NICOLET 6700) were also conducted.

### Electrochemical Measurements

The electrodes were prepared by coating Cu foil with slurries containing the Si/void/SiO_2_/void/C nanoparticles (70%) as active material, a conducting agent (acetylene carbon, 20%), and polyacrylic acid binder (PAA, 10%), dissolved in N-methyl-2-pyrrolidinone. After coating, the electrode was dried under vacuum at 90 °C for 10 h. The loading of active materials for the coin cell testing was about 1.5 mg cm^−2^, and the thickness of the active materials was about 200 μm. The coin-type half-cells were assembled in an Ar-filled glove box. A polyethylene membrane was used as separator, and the electrolyte was 1 M LiPF_6_ dissolved in a mixed solvent of ethylene carbonate (EC) and dimethyl carbonate (DMC) (1:1, v/v). The cells were galvanostatically charged and discharged in the voltage range of 0.01–3 V vs. Li/Li^+^ using a Land Battery Tester (Land CT 2001 A, Wuhan, China) at different current densities at room temperature. Cyclic voltammetry (CV) was carried out on an electrochemical workstation (Chi604e, China) at a scan rate of 0.05 mV s^−1^ in the voltage range of 0.01–3 V vs. Li/Li^+^. The electrochemical impedance spectroscopy (EIS) was performed on a ZAHNER-IM6ex electrochemical workstation (ZAHNER Co. Germany) in the frequency range of 100 kHz to 10 mHz on a cell in as-assembled condition.

## Additional Information

**How to cite this article**: Yang, L. Y. *et al.* Dual yolk-shell structure of carbon and silica-coated silicon for high-performance lithium-ion batteries. *Sci. Rep.* **5**, 10908; doi: 10.1038/srep10908 (2015).

## Figures and Tables

**Table 1:** Comparison of electrochemical properties of silicon with different structures.

**Comparison of electrochemical properties of silicon with different structures**					
**sample**	**current density**	**Capacity (mAh g^−1^) (cycle number)**	**capacity retention**	**mass of active materials**	**thickness of active materials**
3D mesoporous silicon@graphene 16	1 A g^−1^	1200 (200)	89.1%	–	–
silicon@TiO_2-x_/carbon microfiber^19^	200 mA g^−1^	1050(50)	90%	–	–
Si/CNT/C composite^20^	1 A g^−1^	2100 (100)	95.5%	0.6-0.8 mg cm^−2^	–
Si-SiO_2_ core@shell nanowire^21^	420 mA g^−1^	3371 (50)	62%	–	–
Si-SiO_x_ Core-Shell Nanowire^22^	740 mA g^−1^	1910 (100)	95%	–	–
Si@SiO_x_/C Nanocomposite^24^	150 mA g^−1^	1100 (60)	–	–	–
Si-C yolk shell structure^26^	420 mA g^−1^	2800 (1000)	74%	–	–
Silicon@carbon hollow core–shell^28^	50 mA g^−1^	625.3 (40)	76.8%	–	–
Present work	460 mA g^−1^	1147 (430)	83%	1.5 mg cm^−2^	200 μm
